# Genetic Diversity among Rose Rosette Virus Isolates: A Roadmap towards Studies of Gene Function and Pathogenicity

**DOI:** 10.3390/pathogens12050707

**Published:** 2023-05-12

**Authors:** Jeanmarie Verchot, Venura Herath, Ramon Jordan, John Hammond

**Affiliations:** 1 Department of Plant Pathology & Microbiology, Texas A&M University, College Station, TX 77845, USA; 2 Department of Agriculture Biology, Faculty of Agriculture, University of Peradeniya, Peradeniya 20400, Sri Lanka; 3 Floral and Nursery Plants Research Unit, US National Arboretum, United States Department of Agriculture, Agriculture Research Service, Beltsville, MD 20705, USA

**Keywords:** emaravirus, rose rosette virus, negative-strand RNA virus, rose, plant bunyavirus

## Abstract

The phylogenetic relationships of ninety-five rose rosette virus (RRV) isolates with full-length genomic sequences were analyzed. These isolates were recovered mostly from commercial roses that are vegetatively propagated rather than grown from seed. First, the genome segments were concatenated, and the maximum likelihood (ML) tree shows that the branches arrange independent of their geographic origination. There were six major groups of isolates, with 54 isolates in group 6 and distributed in two subgroups. An analysis of nucleotide diversity across the concatenated isolates showed lower genetic differences among RNAs encoding the core proteins required for encapsidation than the latter genome segments. Recombination breakpoints were identified near the junctions of several genome segments, suggesting that the genetic exchange of segments contributes to differences among isolates. The ML analysis of individual RNA segments revealed different relationship patterns among isolates, which supports the notion of genome reassortment. We tracked the branch positions of two newly sequenced isolates to highlight how genome segments relate to segments of other isolates. RNA6 has an interesting pattern of single-nucleotide mutations that appear to influence amino acid changes in the protein products derived from ORF6a and ORF6b. The P6a proteins were typically 61 residues, although three isolates encoded P6a proteins truncated to 29 residues, and four proteins extended 76–94 residues. Homologous P5 and P7 proteins appear to be evolving independently. These results suggest greater diversity among RRV isolates than previously recognized.

## 1. Introduction

Rose rosette virus (RRV) is an enveloped virus with a negative-strand RNA genome and is a member of the genus *Emaravirus* within the family *Fimoviridae* [1]. RRV has seven genome segments [2,3], and all are monocistronic except RNA6, which is bicistronic. RNA1 encodes the putative RNA-dependent RNA polymerase (RdRp), RNA2 the glycoprotein precursor (GPP), RNA3 the nucleoprotein (NP), and RNA4 the movement protein (MP) [4,5]. RNAs 1–3 alone are sufficient for local replication and encapsidation [6]. The RRV genes encoded by RNAs 5 and 7 have unknown functions [7]. RNA6 encodes two proteins known as P6a and P6b. The P6bs of other emaraviruses have been suggested to confer pathogenicity, and it is referred to as an ‘ABC’ protein [8]. The P6b homolog of High Plains wheat mosaic virus (HPWMoV) is reported to have silencing suppressor activity [9,10].

The mutation spectrum within a virus species impacts virus fitness, adaptive responses, antigenic drift, and natural selection. Mutations can offer biological advantages or disadvantages, influence the behavior of a virus population, and can also lead to the accumulation of defective interfering (DI) RNAs [11]. Recent investigations of the population of RRV across North America [12] characterized the population structure of RRV by examining individual genome segments and reported nucleotide identities ranging from 93 to 99%. The analysis of individual genome segments did not reveal genetic changes that could be useful molecular markers to differentiate strains or isolates of RRV across North America [12]. Identifying molecular markers is important to describe the dissemination of virus strains and differential host interactions. Furthermore, identifying various subtypes and subgroups based on amino acid sequence variations is important for characterizing virulence determinants that are linked to different disease etiologies. For segmented RNA viruses, genetic changes derive from mutations, recombination, or segment reassortment. The primary source of mutations is the misincorporation of nucleotides during template copying by an error-prone viral RdRp [13,14]. Transitions are the exchange between purines or between pyrimidines and are more common than transversions, which are the exchange of a purine for a pyrimidine or vice versa [15]. Insertions or deletions, known as indel mutations, are another form of misincorporation of nucleotides. Indels happen during replication by misalignment of the RdRp along the template and most often occur at short repeated sequences or homopolymeric tracts [16]. Recombination and reassortment often occur when virus strains co-mingle in a common host or vector. Within emaraviruses, both recombination and segment reassortment have been reported for fig mosaic virus (FMV) [17] and pigeon pea sterility mosaic virus 1 and 2 (PPSMV-1 and PPSMV-2) [18]. In addition, evidence from HPWMoV supports probable reassortment [19,20,21].

RRV causes considerable economic losses and is a threat to rose production across the USA and Canada. Breeding priorities for rosebushes are to incorporate genetic resistance to RRV alongside other desirable traits. The general descriptions of virus strains or species subgroups are rooted in the categorization of genetic variation that influences the foliar disease pattern, or the nature of its engagement with host genetic resistance. For many virus diseases, there are known strains that elicit mild to severe symptoms, from mild leaf chlorosis or vein clearing to necrosis, and interact with host genetic resistance genes that confer protection against infection. Host resistance is described as extreme (ER) resistance where no symptoms develop, hypersensitive (HR) resistance where local lesions appear in inoculated leaves (usually without systemic infection), or immunity when plants are insusceptible to infection [22]. Plants encode virus resistance (R) genes that can recognize viral protein elicitors to trigger host defenses. The durability of resistance is determined by mutations occurring in the region of the viral elicitor that interacts with the R protein [23]. Virus resistance breaking (RB) strains typically have acquired mutations within the elicitor domains that eliminate R protein recognition to enable successful infection [24,25]. The development of durable control strategies will require understanding regions of the virus genome that show significant genetic variation that can drive virus–host interactions and virus evolution [26].

Common commercial roses are the products of interspecific hybrids or the products of hybrid crossings. For example, tea roses are themselves hybrids: the floribunda rose bush is a cross between the hybrid tea rose and the polyantha rose. Windham et al. (2023), in this special issue of *Pathogens* [27], explain that the major commercial rose cultivars are bred from genetically diverse North American, European, and Asian species or hybrids derived from several *Rosa* taxonomic sections [28,29,30]. Since most commercial varieties are susceptible to rose rosette disease (RRD), breeders are performing replicated trials to identify new genetic sources of RRV resistance, and this requires using a consistent inoculum and introducing good disease pressure to produce symptoms in young rose plants [28,31,32]. Breeding trials depend upon the recognition of common disease characteristics among isolates or strains, accurate and robust diagnostic detection in infected plant samples, and an understanding of the genetic diversity and population structures that influence the emergence of RB strains. This study was undertaken to gain insight into the genetic diversity and the nature of the genetic changes among the complete genomes of sequenced RRV isolates. 

## 2. Materials and Methods

### 2.1. RRV Sequence Database Construction

All previously available RRV sequences were fetched from the National Center for Biotechnology Information (NCBI) Database GenBank (https://www.ncbi.nlm.nih.gov/genbank/, accessed on 5 August 2021) to Geneious Prime software (http://www.geneious.com; accessed on 1 January 2022). Geneious Prime v. 2022.0 and v. 2023.0.4 (Dotmatics Software Co., Bishop’s Stortford, UK) was used to build a database of all RRV genomes and a parallel database of accessions and common names for all reported genome segments was prepared using Microsoft^®^ Excel^®^ (v. 2304) for Microsoft 365. Incomplete genome sequences were discarded from both databases and only the full genome sequences with seven segments were maintained for further analysis. Sequences were aligned using translation align and then translated to validate the start and stop codons for all coding sequences and surrounding untranslated regions (UTRs). All individual genome segment nucleotide sequences were aligned using MUSCLE and trimmed down to the same length represented by the 91 GenBank sequences derived from multiplex amplicon sequencing [12], which did not contain the 16–61 nts from both the 5′ and 3′ terminal ends of each genome segment. This also helped to check for and trim extra bases that have been erroneously added during high-throughput sequencing (HTS). All sequences were named in the Microsoft^®^ Excel^®^ and Geneious Prime databases according to the isolate name reported in GenBank and their reported originating USA state. 

Two new genomes were also included in this study. Samples of rose plant shoots showing severe phyllody from RRD-affected *Rosa* × *fortuniana* plants growing in the US National Arboretum (USNA) National Herb Garden’s “Historic and Species Rose Garden” in Washington DC (isolate named RF-DC) and those from *Rosa* sp. ‘Stormy Weather’ shoots exhibiting typical RRD symptoms in an RRD-resistance field trial in Wilmington, DE (isolated named SW-DE), were collected in 2017 for HTS analysis. Total RNAs were isolated by the CKC (CTAB, KOAc, silica column) protocol (RF-DC sample) [33] or using the MagMAX™ Plant RNA Isolation Kit (ThermoFisher, Waltham, MA, USA)protocol (SW-DE sample) and prepared for HTS on the NextSeq^®^ 500 platform (llumina, San Diego, CA, USA) as described in [34]. Assembly and analysis of the resulting HTS data resulted in contigs representing nearly complete sequences (>99%) of each of the seven RRV genome segments from both isolates (RF_DC and SW_DE, respectively).

### 2.2. Performance of ML Analysis and MAFTT Alignments and Analysis of Sequence Variability and Mutations

Geneious Prime was used to concatenate the nucleotide and amino acid sequences of all seven segments for each virus isolate. Maximum likelihood (ML) analyses were performed using MAFTT alignment and the E-INS-i algorithm; the nucleotide scoring matrix was 200PAM/k = 2, the amino acid scoring matrix was BLOSUM62, and both analyses had a gap open penalty of 1.53. The best-fitting nucleotide and amino acid substitution models with the lowest BIC score were determined using iQTREE v. 1.6.12 [35,36] and then ML trees were imported into FigTree v.1.4.4. (both softwares from http://github.com, accessed on 1 January 2022) and rerooted using manually selected outgroups. PHYML trees were constructed using the amino acid sequences and the VT model. The rate category and gamma distribution parameters were set to 4. 

DnaSP v6.12.03 × 64 (ub.edu/dnasp)was used to perform a comprehensive analysis of nucleotide polymorphisms, haplotype diversity, nucleotide diversity, and neutrality tests [37]. Recombination breakpoints were analyzed using RDP5.0, SplitsTree, and SimPlot++ [38,39,40]. The RDP5.0 package includes the inbuilt methods RDP, GENECONV, BOOTSCAN, MAXIMUM CHI SQUARE, CHIMAERA, SISTER SCAN, and 3SEQ. The analyses were conducted with default settings and events were considered significant if the *p*-values were less than 1 × 10^−5^ in at least five methods in RDP5.0. Then, parsimony-informative amino acid mutations were identified using MAFTT alignments imported into MEGA X software v. 10.1.8 [41]. Trees were annotated in iTOL v6 to display informative mutations [42]. 

## 3. Results

### 3.1. Virus Isolates and Genetic Variants

**Figure 1:**
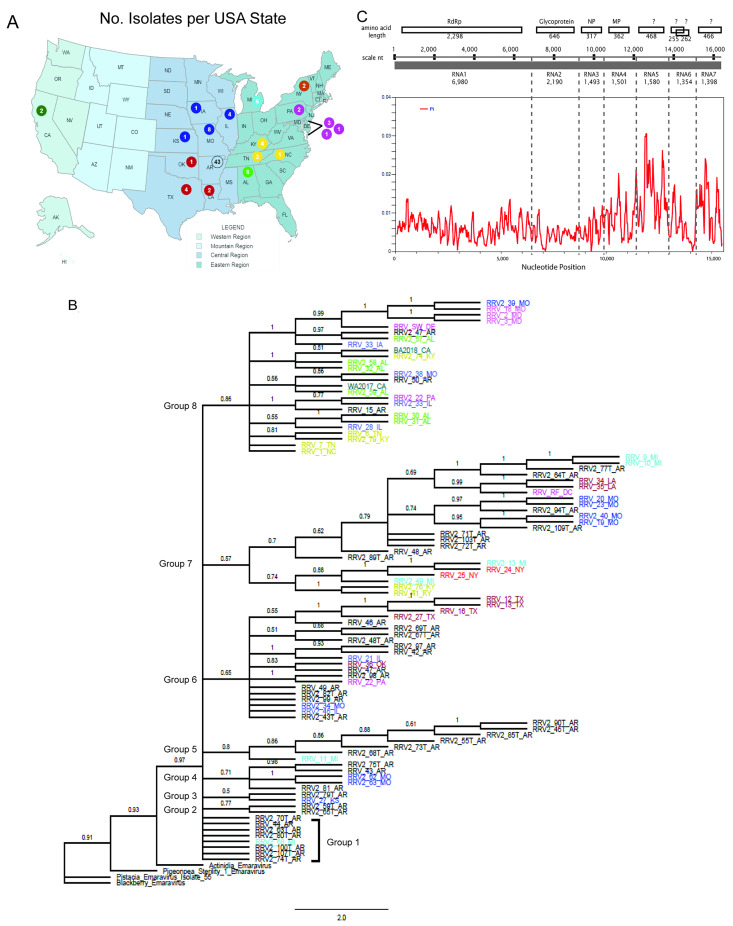
RRV isolates and nucleotide diversity. (**A**) RRV isolates were obtained from 16 states, and one isolate is from Washington DC. The colored circles serve as a color legend for panel C to easily compare the geographical location in the map and color-coded isolates in the phylogenic tree. The number in each colored circle indicates the number of isolates from each state location. Notably, the Delaware, Washington DC, and Maryland isolates are featured on the right side of the map. (**B**) Maximum likelihood (ML) analysis of concatenated genome segments with four emaravirus species as an outgroup. The branch length scale bar is presented at the bottom and branches with values >0.5 are labelled. Tip labels are colored according to the circles in panel (**A**). Groups were assigned to assist the reader. (**C**) Top illustration of the concatenated RRV genome segments. Open boxes represent the open reading frame (ORF), and the amino acid lengths of the encoded proteins are presented. The viral RNA-dependent RNA polymerase (RdRp), glycoprotein (GP), nucleocapsid protein (NP), and movement protein (MP) are identified above the box. Question marks identify genes of unknown functions. The scale below the boxes provides a reference for the RNA segment lengths that are concatenated and identified by the thick gray bar. The nucleotide lengths of each RNA are provided. Dotted lines represent the termini separating each segment. The graph features the nucleotide diversity along the length of the concatenated sequence.

Ninety-three complete genome sequences of RRV were downloaded from GenBank, and two complete genome sequences were obtained by direct sequencing. The locations reported in GenBank are distributed within seventeen USA states (Table S1 and Figure 1A) [12]. There were 43 genomes reported from Arkansas, but we were unable to independently confirm whether the designated location referred to the state location of the research team that deposited the sequences or if this referred to the garden locations that were sampled [43]. The GenBank database reports nine isolates from Alabama and Missouri and between one and four isolates for all other states. The locations of the two isolates from California were reported [44] and their geolocations are noted (BA2018: 35.41228, −119.09160; WA2017: 35.59325, −119.29216). Two additional isolates were from Wilmington, Delaware (39.66507, −75.74429), and Washington, District of Columbia (38.91166, −76.96934) (Figure 1A). These isolates are referred to as RRV_SW_DE and RRV_RF_DC. In this study, we added a suffix USA state abbreviation to each named isolate, and from here onwards, isolate names will be described without the initial ‘RRV’. The isolates’ full names (including ‘RRV’) are given in Supplementary Table S1 (which also includes the GenBank accession numbers for all RRV genome segments) and all figures. Given the low numbers of isolates per state, except for Arkansas, there are unfortunately insufficient data to determine whether the geographic distribution is a factor in the phylogenetic relatedness of these isolates [12]. 

The source host plants for the 91 isolates reported by Katsiani et al. (2020) [12] and the BA2018 isolate from CA [40] were only listed as ‘*Rosa* sp.’. The WA2017_CA isolate was reported from *Rosa* sp. ‘Veteran’s Honor’ (a hybrid tea rose) [40]. The RF_DC was taken from a sample of ‘*Rosa* × *fortuniana*’, which is believed to be a *R. banksiae* × *R. laevigata* hybrid that has nearly thornless canes and is known as a climbing shrub rose grown on fences or walls. The RF_DC isolate was obtained from a plant in the USNA’s National Herb Garden’s “Historic and Species Rose Garden” that was demonstrated by high throughput sequence (HTS) analysis to be coinfected with blackberry chlorotic ringspot virus and rose spring dwarf-associated virus (other viruses to be published elsewhere). The SW_DE isolate was obtained from another climbing rose ‘Stormy Weather’ that was only infected with RRV and was obtained from an RRD-resistance research field. Notably, the reference genome reported in NCBI GenBank is derived from two different isolates and was not included in this study [12]. Another isolate from Oklahoma (NVWA6166361/OK-1) was reported in GenBank in 2021 but was not included in this study because of differences among the noncoding sequences for each segment that led us to be uncertain whether these were complete genome sequences.

ML analysis was performed using concatenated genome segments (Figure 1B). A prior phylogenetic study [8] of the genus *Emaravirus* identified four clades (A, B, C, and D). RRV was grouped into clade A along with actinidia chlorotic ringspot associated virus (AcCRaV), pigeon pea sterility mosaic virus 1 (PPSMV-1), pistacia virus B (PVB), and blackberry leaf mottle-associated virus (BLMaV). The concatenated genomes of these four virus species were used as outgroups. We identified six major groups of isolates based on their relationship to the first node above the root and identified two subgroups, A and B, within the largest group, group 6 (Figure 1B). For example, group 1 has seven RRV isolates from Arkansas and Missouri, which are in the Central Region of the USA (Figure 1C). Group 2 has eight isolates and includes the newly sequenced isolate RF_DE alongside isolates from Arkansas, Louisiana, and Michigan. Group 3 has nine isolates from New York, Michigan, and Kentucky. Group 4 has seven isolates from Arkansas and Missouri. Group 5 has nine isolates, mainly from Arkansas, with one from Michigan. Groups 2 through 5 include isolated genomes from the Eastern and Central Regions of the USA. Group 6 has 54 isolates and is divided into subgroup A from Arkansas, Kansas, Illinois, Missouri, Oklahoma, Pennsylvania, and Texas and subgroup B, which includes isolates from across the USA, including the Eastern, Central, and Western regions. Since commercial roses are typically grown from rooted cuttings and shipped nationally, the distribution of related isolates across geography suggests that some plants may have derived from the same source materials before shipping to different states or become infected by viruliferous mites in the gardens where they were planted. 

To examine the genetic variation across isolates, genome segments were concatenated, extending a length of 15,524 nt. The linked protein sequences consisted of 4847 amino acids (Figure 1C). The statistical estimate of nucleotide diversity (Pi) for RNA segments 1, 2, and 3 ranged from 0.000 to 0.014, which is a small variance among the segments that encode the core replicase and structural proteins (Figure 1C). The nucleotide diversity of RNA segments 4 through 7 ranged from 0.000 to 0.031, representing a broader range of variability, with RNA5 and RNA7 showing the most diversity. The genetic differences among the concatenated genomes were analyzed using DnaSP (Table 1). There are 1648 polymorphic sites (S) and 1703 mutations (ƞ), indicating that there were multiple changes among isolates, and some changes occur at the same nucleotide locations. DnaSP calculated the average pairwise differences among concatenated sequences to be 113 [12]. The overall nucleotide diversity (π) was 0.0073, which is >0.005, indicating low genetic diversity among RRV isolates.

The uneven distribution of polymorphic sites, mutations, overall nucleotide diversity, and nucleotide differences suggests that the mutations are independent. Given that the genome segments vary in size from 6980 nt for RNA1 to 1086 nt for RNA4, the polymerase error frequency among segments may vary and there may be different selection pressures acting on viral gene products. For RNA1 through RNA6, the total number of mutations (ƞ) is greater than the number of polymorphic sites (S), i.e., some mutation sites have different substitutions in different isolates. RNA1 has the greatest number of mutations, 792. RNA3 and 4 have the lowest number of mutations, 83 and 98, respectively. RNA7 has only one more mutation than polymorphic sites (Table 1). The nucleotide diversity (π) values for RNA5 and RNA7 stand out as the highest, 0.0149 and 0.0104, respectively, while RNA2 is the lowest, 0.0060. The molecular diversity patterns were calculated using Tajima’s D, as well as Fu and Li’s D and F statistical tests (Table 1). For the concatenated sequences and each segment, tests were performed using the ‘sliding window’ option, and no positive values were found. The neutrality tests were significantly negative, indicating that the mutation rate is not constant and may be best explained by recent population expansion.

### 3.2. Evidence for RRV Segment Reassortment 

**Figure 2:**
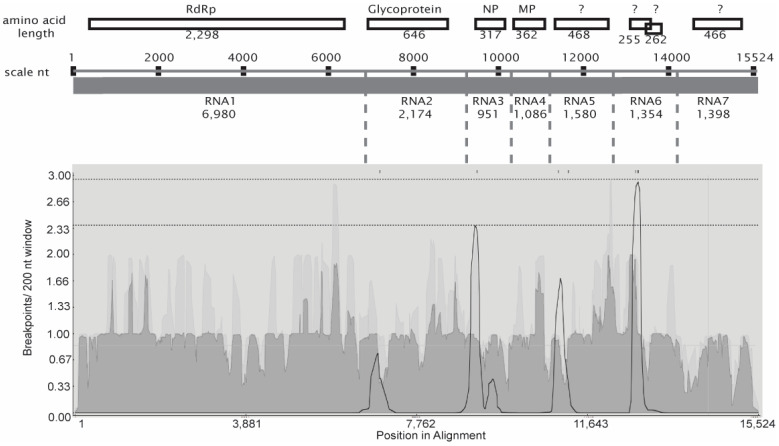
Breakpoint distribution plot presented below the illustrated concatenated genome. The dotted lines across the top of the plot identify the 95% and 99% confidence thresholds. Diagrammatic representation of the concatenated sequences, *p* values, nucleotide lengths, and termini of each segment as in Figure 1, but used here as a reference to understand the breakpoint locations in the outputs.

To assess the potential for reassortment of genome segments, the concatenated sequences were analyzed for recombination breakpoints using RDP5.0, and events were validated using Splitstree and SimPlot++. The recombination breakpoint distribution plot (Figure 2) also identifies potential breakpoints near the junctions of RNA segments. The major and minor parents, as well as the breakpoint positions and the statistical significance for the RDP tests, are provided in Table 2. Three breakpoint positions, 6928, 6982, and 7184, are neighboring the junction of RNA1 and RNA2 in the concatenated sequences. According to this plot, the breakpoints adjoining RNA1 and 2 are not represented with high confidence. The breakpoint position 9134 is at the junction of RNA2 and RNA3. The breakpoint position 12,801 lies near the junction of RNA5 and 6. 

**Figure 3:**
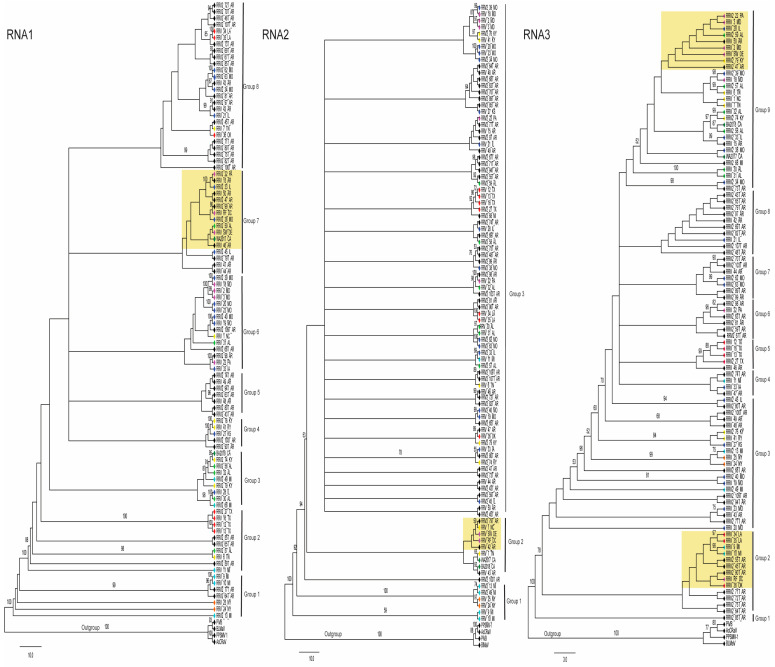
Phylogenetic relationships of RRV RNAs 1, 2, and 3 segments (left to right). The phylogenetic relationships were inferred using the maximum likelihood method (see Section 2). Bootstrap values >50% are shown (10,000 replicates) next to the branches. Branches leading to significant clusters of isolates were assigned a group number to assist in the explanation of the results. The colored diamonds at the branch tips identify the locations as in Figure 1. The trees are drawn to scale. The yellow box surrounds the clusters of isolates that include the two new isolates, SW_DE and RF_DC.

To assess the potential for genetic exchanges involving RNA2 and RNA3, we examined the topology of the phylogenetic relationships between RNA1, 2, and 3. Trees were rooted with the corresponding genome segments of the same virus species used as outgroups in the prior analysis (Figure 3)**.** The branch patterns of these three trees reflect different evolutionary pressures acting on these genome segments. Certain groups of isolates consistently cluster across these trees, such as the four isolates from Texas, 12_TX, 13_TX, 16_TX, and 2_27_TX. Another example is three isolates, 2_13_MI, 24_NY, and 25_NY, which are identified as minor parents in Table 2, meaning that these likely did not arise by segment reassortment. Their RNA1, RNA2, and RNA3 segments are consistently represented as closely related branches, which supports the notion that these isolates did not arise by reassortment of segments involving other isolates. This is contrasted by the three isolates from Maryland known as 2_MD, 3_MD, and 18_MD, which RDP5 predicts arose by segment reassortment. The RNA1s of these three Maryland isolates are in group 6 in the tree (Figure 3) and cluster with three isolates from Missouri, 20_MO, 23_MO, and 2_39_MO. The RNA2s for the Maryland isolates cluster in a subclade at the top of the tree in group 3 with 20_MO, 23_MO, and 39_MO, and two isolates from Kentucky, 41_KY and 76_KY. The branch positions of RNA3 for these Maryland isolates is more distinct with 2_MD located between SW_DE and 50_AR, while 3_MD is between 28_IL and 2_22_PA, and 18_MD is near 2_39_MO, 6_TN, 7_TN, 1_NC, and 2_57_AL (Figure 3). 

**Figure 4:**
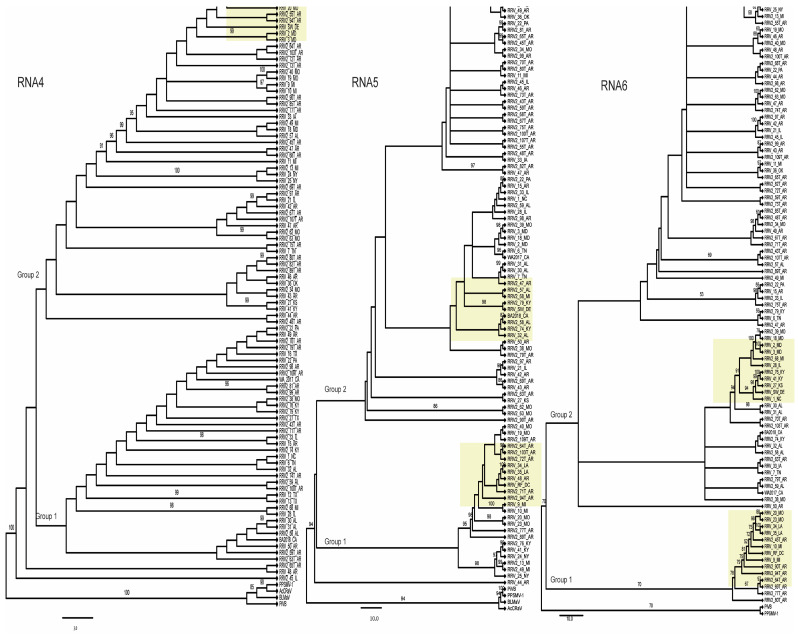
Phylogenetic relationships of RRV RNAs 4, 5, and 6 segments. The phylogenetic relationships were inferred using the maximum likelihood method (see Section 2). Bootstrap values >50 are shown (10,000 replicates) next to the branches. Branches leading to significant clusters of isolates were assigned a group number for reader orientation. The trees are drawn to scale. The yellow boxes surround the clusters of isolates that include the two new isolates, SW_DE and RF_DC. Trees were rooted as before except for RNA6, where there are only two species that can serve as outgroups. The branch patterns of these trees reflect different evolutionary pressures acting on these genome segments.

We report the sequence for two new isolates, RF_DC and SW_DE, which are from the Eastern Region of the USA. RDP5 analysis and the ML trees in Figure 3 and Figure 4 suggest these may have arisen by segment reassortment. To feature these isolates more prominently in the trees the subgroups of branches containing these two isolates are highlighted in yellow in Figure 3 and Figure 4. For RNA1, both new isolates appear in group 7, but for RNA2 at different positions in the tree, in group 2. For RNA3, RF_DC is in group 2, and SW_DE is in group 9. (Figure 3). For RNA4, both fall closer together within group 2 at the top of the tree, while for RNA5 and RNA6, RF_DC is in group 1 and SW_DE in group 2 (Figure 4). Their nearest neighboring isolates also differ between all of the RNAs (Figure 3 and Figure 4).

### 3.3. Insertion and Deletions in RNA6 Occurring along Highly Homopolymeric Tracts

**Figure 5:**
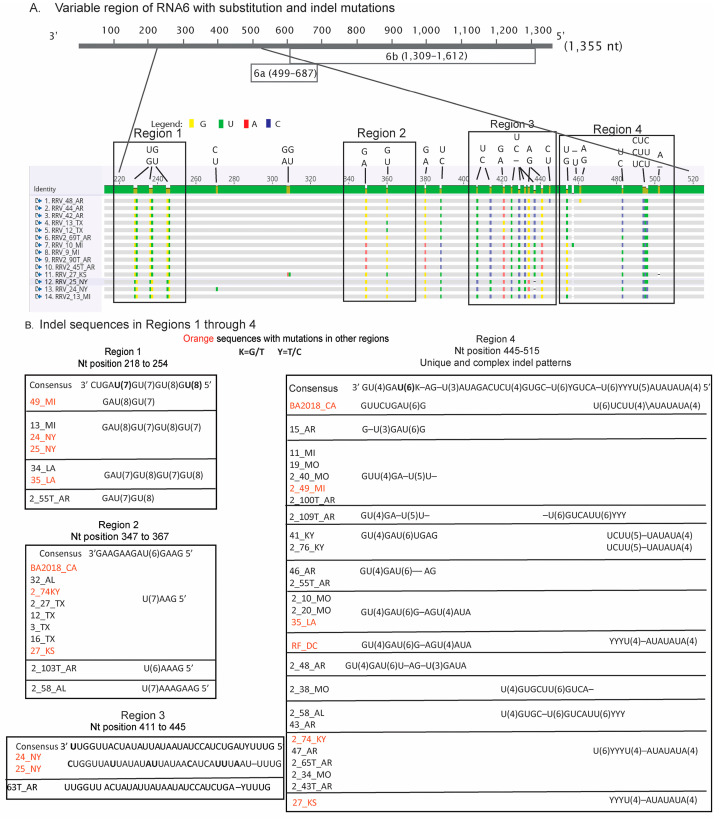
Analysis of RNA6 indel mutations. (**A**) Illustrative outcomes featuring highly variable sequences of RNA6 among non-recombinant genomes. The consensus bar identifies conserved sequences in green and the goldenrod color indicates the locations of substitution or indel mutations in the sequence. The legend represents the colors ascribed to different nucleotide substitutions in the alignment. These changes are also shown in the 1, 2, 3, and 4 boxes above the consensus bar. (**B**) The indel changes in regions 1 through 4 are elaborated. Each box contains the identity of the (non)recombinant genomes associated with each mutation. Some sequences have more than one change in the same region. Sequences with mutations in more than one region are highlighted in orange.

RNA6 encodes two overlapping open reading frames (ORFs) encoding P6a and 6b in the reverse orientation (Figure 5A). The P6a ORF extends from genomic nt position 687 to 499 and is followed by a 499 nt noncoding region at the genomic 3′ end. P6b extends from the genomic 1309 to 612 nt and is preceded by a short 46 nt noncoding region. The RNA6 sequences of 14 parental isolates (determined by RDP5 in Table 2) were aligned (Figure 5A). We identified four regions between nucleotide positions 220 and 520 that are rich in short poly U tracts and have a significant number of nucleotide changes, including indels (Figure 5A). This region is mainly within the genomic 3′ UTR, and also overlaps the end of P6a. The most obvious changes were transitions or transversion mutations occurring inside or near these poly U tracts. The most common changes were G↔ U or C ↔ U. There were a few G ↔ A but no C↔ A changes. Interestingly, most trees show that the 2_13_MI, 24_NY, and 25_NY isolates cluster, and Figure 5B shows that these isolates have the same single-nucleotide deletion in region 1. The two NY isolates (but not the 2_13_MI isolate) have the same single-nucleotide deletion in region 3, and the three isolates have different patterns of C ↔ U substitutions in region 4. 

Substitutions and indel mutations throughout the four RNA6 regions of all 95 isolates were analyzed, and 46 sequences showed changes from the consensus (Figure 5B). In total, we identified single indels in region 1 of seven isolates, which is bracketed by nt positions 218 and 254. There were 10 isolates with changes in region 2 (nt positions 347–367), 3 isolates with changes in region 3 (nt positions 411–445), and 27 isolates with changes in region 4 (nt positions 445–515). Compensatory mutations may exist in other areas of RNA6 outside of these four featured regions. Across all regions, the most obvious changes were in the addition or deletion of a U along a poly U stretch (Figure 5B). Only one sequence, 2_55T_AR, does not have a compensatory mutation in any of the four indel regions, and its P6a and P6b ORFs are the typical lengths. Indel region 2 extends from nt position 347 to 367 and has a single U(6) tract that has an extra U or UAAA in 10 isolates. Region 4 is particularly interesting because it overlaps the translation stop codon for the ‘typical length’ P6a.

**Figure 6:**
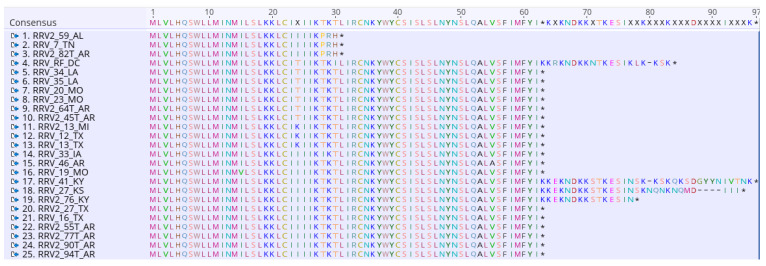
Analysis of twenty-five P6a proteins. Alignment demonstrates that most P6a proteins are 61 amino acids in length and identifies truncated and extended sequences. Amino acid letters are colored to identify similar residues. The * identifies the translation stop codons.

Alignment of the 95 P6a sequences showed that the majority are 61 amino acids in length. Figure 6 presents a snapshot of a larger alignment featuring isolates that deviate from the typical amino acid length of P6a. Three RRV isolates, named 2_59_AL, 7_TN, and 2_82T_AR, encode truncated proteins that are 29 amino acids in length. Four isolates (RF_DC, 27_KS, 41_KY, and 76_KY) encode proteins that extend 75 to 94 amino acids in length. 

### 3.4. Amino Acid Changes within RRV Coding Sequences and Proteins

Reassortment between isolates can lead to the exchange of gene segments between viruses that can result in progeny viruses that are genetically distinct and can be associated with antigenic shift and the emergence of new pandemic strains. Understanding the patterns of amino acid changes is important to the identification of virus isolates that may be distinguishable by serological methods or virulence on different hosts. The core virion component proteins are RdRp, GPP, and NP, and these contain 31, 20, and 2 parsimony-informative residues, respectively. MP, P5, P6a, P6b, and P7 have between 3 and 29 informative changes (Table 3). 

**Figure 7:**
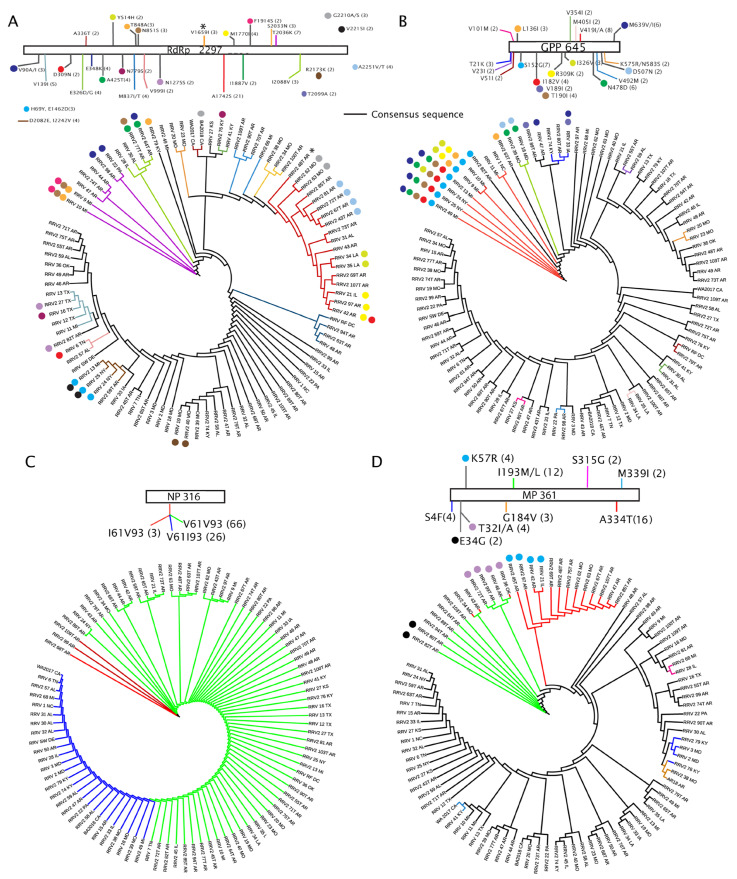
ML trees of sequences encoded by RNA1 through RNA4 with parsimony-informative amino acid changes. Open bars at the top of each panel provide a legend for each tree, the name of the protein, and its amino acid length. (**A**) RdRp, (**B**) GPP, (**C**) NP, and (**D**) MP. The approximate locations of amino acid changes are represented along the bar as colored lines or colored circles. The consensus amino acid and its position are listed followed by the substituted amino acid. The numbers in parentheses identify the number of isolates with this amino acid change from the consensus. The colored branches in the phylogenic trees correspond to the colored lines in the legends. Colored dots along the ML trees also identify amino acid changes represented by the same dots in the legends. Black branches without associating dots represent the isolates that conform to the consensus sequences.

We mapped single amino acid variations to the phylogenetic trees derived from ML analysis of 95 isolates and used color coding of the tree branches to represent the most common changes from the consensus. Branches represented as black lines are identical to the consensus sequence. Various colored dots were used to identify additional amino acid changes that could not be represented by the colored branches (Figure 7A–D). As expected, the trees show clustering of amino acid positions with the same variant residues among closely related isolates. The largest number of amino acid changes (expected for the NP) are distributed at the base of each tree, while more distant branches mainly represent the consensus sequence. 

Amino acid changes that represent a clade (and potentially with a few outliers which share the same residue but fall into other clades) are defined here as a ‘group’. Isolates sharing parsimony-informative sites but not predominantly in a single clade are not defined as a ‘group’ but are mentioned in other terms. For example, seven isolates at the base of the RdRp ML tree share a common T_2036_K change (magenta branches). Added mutations divide the branches in this group in a manner that suggests there are three diverging subgroups. The first subgroup has only the single T_2036_K mutation, the second subgroup of two PA and AR isolates has an additional V_90_A/I, and the third subgroup has two MI isolates with additional T_848_A, N_851_S, and F_1814_S (Figure 7A). There is a neighboring clade of four isolates that appear to be defined by I_2088_V (green branches), and there are similar groups of two to five isolates with at least one common amino acid change. One large group of 21 isolates (red branches) shares a common A_1742_S substitution (Figure 7A). This S_1742_ group could be further characterized as having four subgroups: two MO isolates that have a G_2210_A, four AR isolates that have an A_2251_V/T mutation, two LA isolates with Y_514_H, and three IL and AR isolates with M_1170_I mutations, of which the 42_AR isolate has an additional D_309_N mutation. 

This ML tree further demonstrates that isolates from the same state may not be closely related. This is exemplified by the distinct grouping of MO isolates based on four amino acid changes: V_1659_I, S_2033_N, A_1742_S/G_2210_A, and R_2173_K. The 20_MO, 23_MO, and 2_48T_AR share V_1659_I; 34_MO, 38_MO, and 2_100T_AR share S_2033_N; and 19_MO and 2_40_MO share R_2173_K. Another example is the four MI isolates. The 9_MI and 10_MI isolates pair together with four common changes mentioned previously, while the 11_MI isolate clusters with four TX isolates sharing a common V139I. Isolate 13_MI clusters with two NY isolates and 59T_AR with a common pair of mutations, D_2082_E and I_2242_V. The 13_MI and two NY isolates also share another two mutations, H_69_Y and E_1462_D, but only 13_MI and 25_NY share the V_2215_I mutation.

The ML tree of the GPP sequences shows clustering of amino acid changes among isolates at the base of the tree, but the ordering of branches does not match the RdRp ML tree (Figure 7B). There are twenty parsimony-informative amino acid changes occurring in 27 isolates (Table 3), and 8 of these isolates have a V_419_I/A change highlighted as red branches in the tree (Figure 7B). The next most abundant mutations are M_639_V/I and N_478_D, each occurring in six isolates. Isolates with the V_419_I/A change have between zero and six other substitutions, including S_152_G, I_182_V, T_190_I, I_326_V, N_478_D, and/or M_639_V/I. Two isolates from MO have the V_354_I and N_478_D changes. One isolate named 47_AR has the M_639_V/I mutation alone, and another isolate, 33_IA, has two additional mutations, T_21_K and V_189_I. Isolates retaining the V_419_I/A substitution are primarily from NY and MI, with only one isolate from NC. Most amino acid changes occur at the base of the tree, with only six substitutions occurring in pairs of distant isolates (Figure 7B). The NP tree can be described based on two amino acid positions creating three type groups, of which 66 isolates have V_61_ and V_93_, 26 isolates have V_61_ and I_93_, and 3 isolates have I_61_ and V_93_ (Figure 7C). 

The only described MP is encoded by RNA4, and it is not currently known if there are other movement-associated proteins encoded by other viral genome segments. Across isolates, the viral MP has nine parsimony-informative amino acid changes (Table 3). As for the GPP, most informative changes occur in isolates that reside at the base of the ML tree. There are twelve isolates with the I_193_M/L change (green branches) and sixteen isolates with the A_334_T change (red branches) (Figure 7D). Notably, there are three isolates represented by green and red branches with both I_193_M/L and A_334_T changes (2_35_MO, 43_AR, and 36_OK). These M/L_193_ and T_334_ groups have deeply rooted branches and are adjacent in the ML tree. Among the I_193_M/L isolates are two isolates with an E_34_G mutation and a cluster of four isolates with a T_321_A mutation (Figure 7D). Among the T_334_ group are four isolates with a K_57_R mutation. 

**Figure 8:**
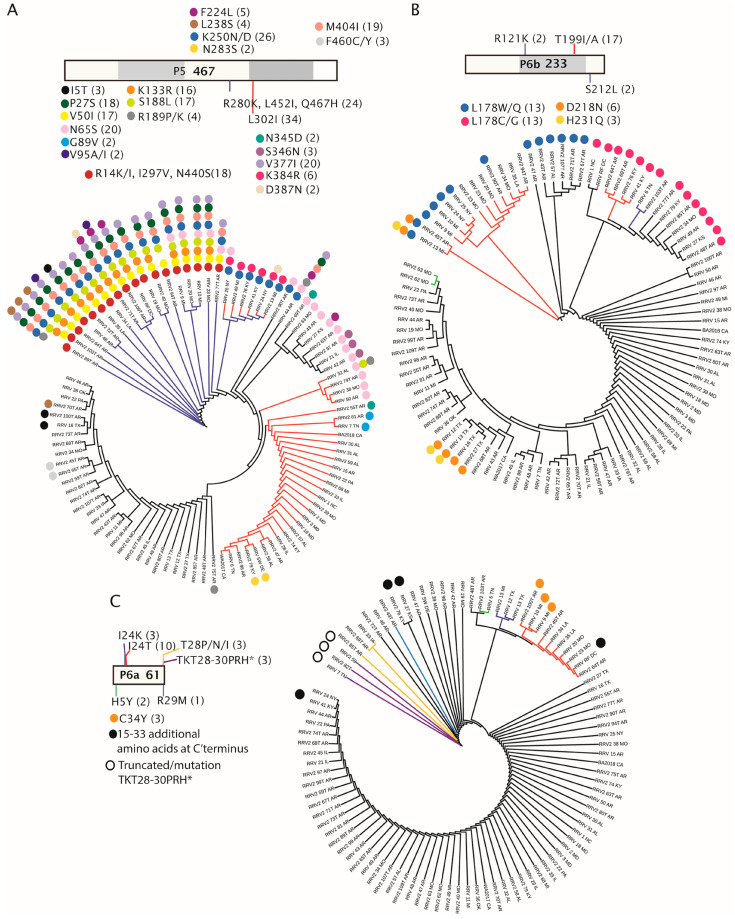
ML trees of sequences encoded by RNA5 and RNA6 with parsimony-informative amino acid changes. Open bars at the top of each panel provide a legend for each tree, the name of the protein, and the amino acid length of the protein. These open bars are divided with grey boxes to represent subdivision along the sequence of 100 amino acids. (**A**) P5, (**B**) P6b, (**C**) P6a. The amino acid changes are listed in order below each subdivision to feature their locations along the sequence. The estimated locations of amino acid changes are represented along the bar as colored lines or colored circles. The numbers in parentheses identify the number of isolates with this amino acid change from the consensus. The colored branches in the phylogenic trees correspond to the colored lines in the legends. Some isolates are represented by two color branches, indicating that two mutations define that cluster. Colored dots along the ML trees also identify amino acid changes represented by the same dots in the legends. Black branches without associating dots represent the isolates that conform to the consensus sequences.

P5 and P7 are 467 and 465 amino acids in length, respectively, and were recently reported to be homologs of unknown function [8]. P5 and P7 have accumulated 29 and 23 parsimony-informative changes, respectively, which are an abundance of changes compared to other viral proteins and amino acid changes (Table 3). The ML tree composed of P5 sequences (Figure 8A) has four potential isolate groups. The first group of 24 isolates (blue branches) is defined by the combination of R_280_K, L_452_I, and Q_467_H substitutions. A second group of 36 isolates is represented by L_302_I (red branches). These two groups share six isolates, which are represented in Figure 8A by blue and red branches indicating that they share the R_280_K, L_452_I, Q_467_H, and L_302_I changes. A subgroup of the K_280_/I_452_/H_467_ group also has the L_302_I change (blue plus red branches). Isolates lacking these mutations are represented by black branches. Eighteen members of the K_280_/I_452_/H_467_ group have the combination of R_14_K/I, I_297_V, N_440_S (represented by deep red dots), and K_250_N/D (royal blue dot) substitutions (Figure 8A). This K_280_/I_452_/H_467_ group also has between four and eight additional changes. Six isolates representing the combination of R_280_K, L_302_I, L_452_I, and Q_467_H substitutions (blue plus red branches) have two additional amino acid changes, K_250_N/D and K_384_R. These six isolates are from NY, MI, and KY. K_250_N/D is conserved with the isolate group represented by the blue branches. Regarding the I_302_ group, there are nine isolates with additional amino acid changes other than the previously mentioned K_250_N/D and K_384_R (Figure 8A). Interestingly, there are 20 isolates with N_65_S, which are distributed among isolates in each of the four type groups represented by colored or black branches in the tree (Figure 8A). Four unique amino acid changes that occur only in isolates represented by black branches include N_283_S, F_460_C/Y, N_345_D, and S_346_N (Figure 8A). The N_283_S is also present in two isolates represented by red branches which have the L_302_I change: 2_79_KY and SW_DE. The N_345_D change also occurs in 2_58_AR, which is also represented by a red branch identifying the L_302_I change. 

Interestingly RNA6 encodes two overlapping proteins, 6a and 6b, and the order of branch isolates in these trees is different. P6a and P6b have three and seven informative amino acid changes (Table 3 and Figure 8). The accumulation of certain mutations in clusters along the phylogeny tree are also different, suggesting different host-selective pressures are acting on these proteins (Figure 8B,C). P6b has no parsimony-informative changes from the consensus sequence in the first 100 amino acids. All changes appear closer to the C-terminus of the protein (Figure 8B). The P6b ML tree shows seventeen isolates with a T_199_A mutation, which are highlighted as red branches. Interestingly, twenty-six isolates have mutations at L_178_. Of these 26 isolates, there are 13 with a W/Q change and 13 with a C/G change, and these occur at the base of the tree. Two deeply rooted branches representing isolates with the T_199I_A (red branches) include six isolates with the L_178_W/Q mutation. Two isolates at the exact base of the tree have T_199I_A, L_178_W/Q, D_218_N, or H_231_Q (Figure 8B). The D_218_N and H_231_Q mutations also occur in a cluster of four isolates from TX, which are more distant in the tree. Five isolates with the T_199_I/A mutation also have the L_178_C/G mutation, and two have an R121K mutation with or without the C/G change at position 178 (Figure 8B). The P6a proteins in this study are more often 61 amino acids in length but seven isolates are either truncated or extended. Three isolates have three neighboring amino acid changes, TKT_28–30_PRH, followed by a stop codon (*), and these are the shortest 6a proteins among the ninety-five isolates (Figure 8C, Figure 6). There are three isolates with a single change at T_28_ that is P/N/I and is not followed by a premature stop codon. The most common change is I_24_T, occurring in 10 isolates that have shallow branches. There is one isolate with R29M, two with H_5_Y, and three isolates from MI and TX with I_24_K mutations. Four isolates extend 15 to 33 amino acids beyond the most common 61 amino acid length (Figure 8C, Figure 6).

**Figure 9:**
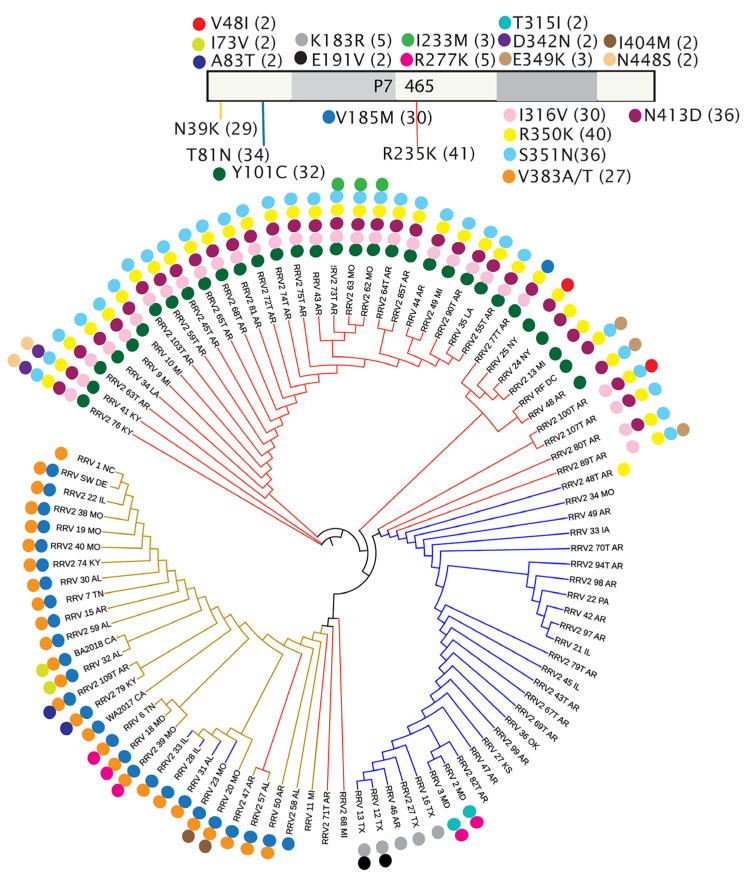
ML trees of P7 sequences with parsimony-informative amino acid changes. Open bars at the top provide a legend, as shown in the prior Figure 7 and Figure 8, the protein’s name, and amino acid length. Some isolates are represented by two color branches, indicating that two mutations define that cluster.

P7 has a complex set of variant residues that are distinct from the changes reported in P5 (compare Figure 8A, Figure 9), suggesting that they evolved separately. Three major divisions of the P7 tree are influenced by 10 informative amino acid changes that occur in 29 to 41 isolates (Figure 9). The most notable substitutions in the ML tree are the R at position 235 in 54 isolates and K at the same position in 41 isolates. The K_235_ group, which is the first major group at the base of the tree, includes 36 isolates that also have changes represented by Y_101_C, I_316_V, R_350_K, S_351_N, and N_413_D. Two isolates at the base of the tree, 41_KY and 76_KY, have two additional mutations that separate them from other members of the K_235_ group, which are D_342_N and N_448_S (Figure 9). There are nine K_235_ isolates that extend from deep branches but lack one or more of the Y_101_C, I_316_V, R_350_K, S_351_N, and N_413_D changes (Figure 9). A second major division of the tree is represented by blue branches in Figure 9, and we identify these isolates for the N at position 81. The N_81_ group has few other changes. It is interesting to note that Y_101_ only co-occurs with T_81_ and that N_81_ never occurs with C_101_ in the same isolate, suggesting that these amino acid positions influence each other. Only eight isolates of this N_81_ group have additional differences from the consensus sequence, and 2_48T_AR has a K_350_, which is more commonly seen in the K_235_ group. The most distant branches of the N_81_ group include two isolates from MD, four isolates from TX, and one isolate from AR (46_AR). The MD isolates have two amino acid changes, R_277_K and T_315_I. The other five isolates from TX and AR have K_183_R, and two TX isolates have E_191_V. 

There are two isolates with K_235_ at the base of the next cluster group that lack any other change, 2_71T_AR and 2_68_MI. This next cluster group is largely defined by K_39_. The first member of this group, 11_MI, has both K_235_ and K_39_ changes. Two isolates in this K_39_ group also have K at position 235 alongside two additional changes known as V_383_A/T or V_185_M. Most isolates in this K_39_ group have one or both V_383_A/T or V_185_M, further defining this group. It is interesting to note that K_39_ can appear in isolates with either N_81_ or K_235_, while these two changes do not occur in the same isolates (Figure 9). 

## 4. Discussion

This study presents evidence of genetic variations across 95 known isolates of RRV having full genome sequences available, in the form of transitions, transversions, indels, recombination, and reassortment. Most often, viral strains are reported based on geographic distribution and rate of change over time according to the molecular clock hypothesis. Given that most of the RRV isolates were obtained around the same time and the greatest number (42 of 95) of isolates are reported to be from Arkansas, we were limited in our ability to examine evolutionary processes influenced by space and time [45,46,47]. Therefore, we examined 95 complete RRV genomes to assess the genetic diversity across all RNA segments. The initial analysis shown in Table 1 reveals relatively high nucleotide conservation across isolates, as previously reported [12]. The negative D and F tests support the hypothesis of recent RRV population expansion. Table 1 presents ƞ, π, and K values which point to nucleotide differences among segments, suggesting that different selection pressures may be acting on each segment. As expected of an error-prone polymerase [48], the mutations were more abundant in the larger RNA1 than in the shorter segments, such as RNA5, RNA6, and RNA7, that range between 1000 and 2000 nt in length. 

While all RNA segments had examples of transition and transversion mutations [15], RNA6 presented a significant number of indel mutations occurring in the 3′ genomic noncoding region. RNA6 encodes in the antigenomic sense two short ORFs, P6a and P6b, which overlap by 76 nt in different reading frames. The 3′ genomic noncoding region is unusually long, extending ~500 nt, and has several poly(U) sequences. Several studies have reported that homopolymeric tracts can cause mutations to occur during replication caused by slippage of the replication machinery [16,49,50]. The genomic 3′ noncoding region of RNA6 might be a hotspot for variation resulting from polymerase errors due to misalignment, slipping, or stuttering of the RdRp during replication [47]. Perhaps RNA secondary structure in this region may influence the mutation rate relative to other genomic regions. Figure 5 shows that some indels occur alongside other substitution mutations or other indels, suggesting that compensatory mutations are occurring as a molecular rescue mechanism. It is worth speculating that indels may be associated with a fitness cost and that secondary mutations during evolution provide solutions contributing to adaptation. Remarkably, seven P6a proteins among the 95 sequences have truncations or extensions at the C-terminus. Since the functions of P6a or P6b are currently unknown, we cannot yet speculate on the biological role of these proteins or the impacts of these amino acid changes. However, it is known that the environment and the host genetic background often shape adaptive mutations in viral genomes. The acquisition of new hosts can impact virus fitness and mutations may arise to improve survival in heterogeneous environments [51,52,53]. Most of the RRV genome sequences used in this study were reported in GenBank and the rose cultivars were not identified in the publication or online materials, therefore, little is known about the host genetic background [12]. Given that commercial roses can be diploid, triploid, or tetraploid, it is reasonable to assume that the RRV isolates reported in GenBank were recovered from rose varieties whose diverse genetic backgrounds likely encourage virus adaptation and host range expansion [54,55,56,57]. Until now, there is no information concerning quasi-species population structure or real estimates of heterogeneity in natural populations for RRV. Such studies will be necessary to better understand the selection pressures that specifically act on RNA6 sequences. 

It is important to address the possibility that some mutations might represent errors in Illumina sequencing [58,59]. Regarding indels in RNA6, the occurrence of compensatory mutations that ensure the integrity of most RNA6 sequences suggests that these are naturally occurring mutations. Unfortunately, mutations can also be induced due to sequencing errors associated with the Illumina technology, which was used for generating the RRV reference genome in 2011 [2]. For example, the NCBI reference genome for RRV RNA1 (HQ871942.1) has a single adenine inserted at position 53, which is adjacent to a natural poly(A) sequence, and a T(10)CT(2) sequence at nucleotide position 1133–1146 that are not found in any of the other 95 isolates that were used in this current study [2,6]. The single adenine insertion significantly alters the position of the translational start codon, causing a 21 amino acid truncation near the endonuclease domain [6], and is another reason NCBI sequences HQ871942-HQ871945 were not used in this study. Ninety-one of the other sequences used in this study were collected in 2016 and were generated using Illumina NextSeq500 or HiSeq3000 devices, which have been reported in test studies to have low insertion and deletion error rates [58]. Two original sequences, RF_DC and SW_DE, were reported here and obtained in 2021, also using this improved Illumina technology (see Section 2). 

The ML phylogenetic tree of the concatenated genomes of all 95 isolates shown in Figure 1 represents the diversity due to all three factors contributing to the evolution of RRV, namely mutation, recombination, and genome segment reassortment. In contrast, the RDP5-identified apparent recombination breakpoints for some isolates approximately at the junctions of RNA1 and RNA2, between RNA2 and RNA3, and between RNA5 and RNA6, suggest genome segment assortment as previously reported for FMV, PPSMV-1, PPSMV-2, and HPWMoV [17,18,19,20,21]. Other breakpoints identified by RDP5 indicate recombination within individual genome segments in various isolates.

The variations in numbers and constituent members of groups in the ML phylogenetic trees of the individual RNA segments (Figure 3 and Figure 4) more clearly show the multiple impacts of genome segment reassortment and recombination within genome segments, exemplified by the varying positions of new isolates RF_DC and SW_DE in the topology of the trees in Figure 3 and Figure 4. It has been demonstrated that identifying and removing recombinant isolates of turnip mosaic potyvirus (TuMV) prior to phylogenetic analysis yielded similar tree topologies from three distinct phylogenetic methods (ML, maximum parsimony, and neighbor joining), thus separating the results of hierarchical evolution from the results of reticulate evolution and correlating well with host adaptation of the non-recombinant isolates [60]. Notably, TuMV has a monopartite genome, so unlike RRV, TuMV is not subject to the effects of genome segment reassortment—and in RRV the combination of recombination and reassortment affects a high proportion of the isolates examined.

Most recombination breakpoints that were identified in the concatenated sequences were located near the junctions of noncoding regions between segments. Recombination and reassortment often occur when virus strains co-mingle in a common host or vector. Recombination and segment reassortment have been reported previously for FMV [17], PPSMV-1, and PPSMV-2 [18]. In addition, the occurrence of two distinct copies of RNA3 in some but not all isolates of HPWMoV, and three phylogenetically distinct clusters of RNA3, are strongly supportive of reassortment [20,21]. The HPWMoV RNAs 1 to 3 are most closely related to those of Palo Verde broom virus, and its RNA4 more closely related to those of Ti ringspot-associated and common oak ringspot-associated viruses [19]. Recombination breakpoints were detected inside RNA1, RNA2, RNA4, and RNA5 of PPSMV-1 and -2, revealing the potential recombination among isolates as well as interspecies recombination. Reassortment of RNA4 and RNA6 was also shown to occur between these two species [18]. 

Figure 7, Figure 8 and Figure 9 explore the amino acid changes occurring in viral-encoded proteins in a virus population infecting a single host, roses. Genetic variation, resulting in amino acid changes, is important for adaptation to a new host or vector genotypes. Unfortunately, when these sequences were reported there was no information about host genotypes or vector populations. Future work will need to examine the interaction of virus and host/vector genotypes to understand the influence of host genotype on virus fitness. Among all the viral proteins, the NP has the least number of changes, suggesting that it might be subjected to a stringent bottleneck or have few interactions with host proteins to require adaptive mutations. The NP most likely has less influence than other viral proteins on host range expansion [61]. It is worth speculating that a combination of changes in the GPP and NP might influence particle dimensions. It is also reasonable to consider that the diversity of GPP variants may be important in influencing receptor interactions involving plant cells and insect vectors, playing a key role in horizontal virus transmission [62,63].

Although rose rosette disease (RRD) was first reported in Canada in 1940 [64], and then in Wyoming in 1941 and California in 1943 [65], and the vector *Phyllocoptes fructiphilus* was reported from the wild *R. californicus* in California [66], for many years RRD was primarily reported from populations of the introduced invasive weedy species *R. multiflora* (multiflora rose) [67,68]. As early as the late 1950s, RRD had been reported to cause significant problems in a rose breeding program in Nebraska in proximity to thickets of wild roses including *R. woodsii* and *R. suffulta* (syn. of *R. arkansana* var. *suffulta*), and also plants of the naturalized species *R. rubrifolia* (syn. of *R. glauca*) and *R. eglanteria* (syn. of *R. rubiginosa*), from all of which *P. fructiphilus* was also obtained and demonstrated to transmit RRV [69]. Despite this early report of the effects of RRV on a rose breeding program, the emergence of RRV as a significant problem in cultivated roses broadly coincided with the spread of RRD from the western to the eastern United States primarily through naturalized stands of *R. multiflora* [70], and subsequently the increased popularity of landscape roses planted in home gardens, as well as in large groupings on commercial properties and in highway medians [71]. It is probable that the expansion into commercial cultivars occurred via the wind-blown distribution of mites from wild populations of *R. multiflora* followed by the significant early adaptation to different commercial rose hybrids over a relatively short period, and by a slower rate of further adaptation as the virus became further distributed among additional cultivated rose hybrids with different genotypes. Interestingly, different relationships among many of the isolates occur across the phylogenetic trees. Both sequence adaptation and genome segment reassortment appear to be important in the evolution of RRV.

The absence of any clear phylogenetic relationship between the geographic distribution of the sequences examined is consistent with the observations of Katsiani et al. (2020) [12] and may reflect the production of different rose cultivars by growers in multiple states and subsequent distribution within the trade. While local spread among varieties is likely to occur after planting in the landscape, some isolates (and *P. fructiphilus*) have probably been distributed to several states in dormant asymptomatic plants. No clear correlations can be drawn in the absence of cultivar and nursery origin data for most of the isolates for which sequences are currently available. 

Virus pathotypes were described long before they could be associated with specific sequence differences, e.g., different soybean varieties are used to classify soybean mosaic virus pathotypes [72], or common bean varieties to differentiate bean common mosaic/bean common mosaic necrosis virus pathotypes [73]. Such pathotype differences are attributed to amino acid variations affecting the disease phenotype. Other amino acid changes have been used to describe virus pathotypes that can overcome disease resistance conferred by R genes in a gene-for-gene model or resistance conferred by quantitative trait loci (QTL) [25,74]. Key to the gene-for-gene model of resistance is that the pathogen has the capacity to evolve resistance-breaking pathotypes in a plant–virus coevolution scheme. Examples among positive-strand RNA viruses include tobamoviruses in pepper, potyviruses and potexviruses in potato, cucumoviruses in cucurbits, and other virus–host pathosystems where mutations in viral proteins impair their recognition by R proteins resulting in resistance-breaking strains [25,74,75,76,77]. Quantitative resistance to virus infection relies on QTLs decreasing pathogen virulence by reducing infection efficiency or modulating major resistance genes. Surprisingly, P5 has 29 and P7 has 23 parsimony-informative sites, which are comparable to the RdRp and GPP. P5 and P7 have been suggested to be homologs that arose by gene duplication, and it is important to note that the parsimony-informative amino acids in these proteins are quite different within an isolate, suggesting that they may be subjected to different selection pressures. Given the number and complexity of mutations in P5 and P7, it is reasonable to assume that the viral populations undergoing plant/vector bottlenecks do not influence the accumulating variants among these two proteins of unknown function. Since RRV isolates derive from commercial roses that are subjected to breeding processes to incorporate simple genetic traits or QTLs, it is worth considering the possibility that the genetic changes in the P5 and P7 proteins may be influenced by the host genetic background [25]. It is arguable that the heritable diversity of the P5 and P7 proteins may also be important for virus adaptation to changing environments. Given that accumulating mutations are often associated with resistance-breaking (RB) variants or increasing virus–host compatibility with host susceptibility factors, it is worth considering that P5 and P7 proteins are important virulence determinants. 

RRV P6b has been identified as an ‘ABC protein’ with homologs in three of four proposed clades of emaraviruses [8], which may have RNA-silencing suppression activity and be involved in pathogenicity [8,9,78]. It will therefore be of considerable interest to compare the pathogenicity of RRV isolates differing in the length and amino acid sequence of P6a, or by substituting RNA6 variants of P6a or P6b length into the infectious clone [5,6].

Researchers who study molecular factors influencing R protein accumulation and the expression of disease resistance have shown that molecular chaperones, the ubiquitin-mediated proteasome systems, or post-translational modifications influence the accumulation and function of proteins and therefore influence the efficiency of resistance responses. It is well documented that molecular chaperones, the proteasome systems, and post-translational modifications can also influence virus accumulation. Thus, the host genetic background that influences genetic resistance can also drive the evolution and emergence of RB isolates. The current analysis of mutations in RRV isolates suggests that there may be a clustering of mutations that could be determinants of subtypes or pathotypes. Unfortunately, since the host source genotypes are unknown for the 92 isolates studied here, determining potential virus pathotypes based on genomic sequence variance would be premature. Further research is needed to examine the biological properties of both the host genotype and virus isolates relative to disease severity, RNA accumulation, or resistance breaking to clearly categorize isolates into pathotypes. 

## Figures and Tables

**Table 1:** Analysis of polymorphic sites and mutations for concatenated and individual RRV segments (all isolates).

Sequence	N	S	ƞ	π	K	Tajima’s D *	Fu and Li’s D **†	Fu and Li’s F **
Concatenated	95	1648	1703	0.0073	113	−2.2586	−4.4978	−4.0345
RNA1	95	769	792	0.0060	42	−2.4870	−4.2207	−4.1514
RNA2	95	218	221	0.0043	9	−2.6536	−4.4239	−4.3939
RNA3	95	79	83	0.0073	7	−1.8977	−4.6653	−4.2174
RNA4	95	95	98	0.0080	9	−1.8117	−4.4080	−3.9841
RNA5	95	209	223	0.0149	239	−1.5531	−3.6656	−3.3067
RNA6	95	146	154	0.0069	9	−2.3190	−4.9792	−4.6142
RNA7	95	133	134	0.0104	14	−1.4900	−3.8783	−3.4378

N, number of genomes; S, total number of polymorphic sites; Ƞ (Eta), total number of mutations; π, nucleotide diversity estimated by the average pairwise differences between all sequences and based on all sites; K, average pairwise nucleotide differences; * *p* < 0.01; ** *p* < 0.02; † test using a sliding window length of 100 and step size of 25.

**Table 2:** RRV reassortment detected with RDP and SimPlot++ *.

Minor Parent	Major Parent	Breakpoint Positions	RDP Analysis (RGCMBSPL3) **
RRV_12_TX and RRV_13_TX	RRV_9_MI and RRV_10_MI	1–9136	6.58 × 10^−13^ to 1.36 × 10^−54^
RRV2_13_MI, RRV_24_NY, and RRV_25_NY,	RRV_44_AR and RRV_48_AR	6982–12,801	5.68 × 10^−5^ to 2.09 × 10^−45^
RRV_27_KS, RRV_42_AR, and RRV2_69T_AR	RRV_9_MI and RRV_10_MI	6928–9134	3.97 × 10^−9^ to 5.82 × 10^−20^
RRV_42_AR	RRV2_45T and RRV2_90T	7184–9134	1.18 × 10^−5^ to 3.91 × 10^−12^

* RDP output was validated by SplitsTree, SimPlot++, and BOOTSCAN using the Kimura 2-parameter distance model with a window length of 1000 and step of 200. ** Analysis was performed on the concatenated alignment of all seven segments using the RDP5 v. 5.34 software. Methods supporting significant recombination or reassortment signals for five or more tests are indicated with the range of significance (*p* values) obtained: R (RDP), G(GENECONV), C (Chimaera), M (MaxChi), B (BOOTSCAN), S (SISCAN), *p* (PhylPro), L (LARD), and 3 (3SEQ).

**Table 3:** Amino acid sequence variation across coding regions of 95 isolates.

Sequence	Amino Acid Length	Parsimony-Informative Amino Acids
RdRp	2297	31 (1%)
GPP	645	20 (3%)
NP	316	2 (0.6%)
MP	361	9 (2%)
P5	467	29 (6%)
P6a	61–94	3 (≤0.05%)
P6b	233	7 (3%)
P7	465	23 (5%)

## Data Availability

Not applicable.

## Supplementary Materials

The following supporting information can be downloaded at: https://www.mdpi.com/article/10.3390/pathogens12050707/s1, Supplementary Table S1: List of RRV accessions used in this study.
